# Effects of self-monitoring physical activity with wearable activity trackers on perceived joint function and health-related quality of life in people with hip and knee osteoarthritis: a secondary analysis of a cluster-randomised clinical trial

**DOI:** 10.1186/s12891-024-08238-8

**Published:** 2025-01-09

**Authors:** Elin Östlind, Frida Eek, Kjerstin Stigmar, Eva Ekvall Hansson

**Affiliations:** 1 Department of Health Sciences, Faculty of Medicine, Lund University, Box 117, Lund, 221 00 Sweden; 2 Ear-Nose and Throat Department, Skåne University Hospital, Lasarettsgatan 21, Lund, 221 85 Sweden

**Keywords:** Hip osteoarthritis, Knee osteoarthritis, Physical activity, Wearable activity tracker, Joint function, HRQoL

## Abstract

**Background:**

Osteoarthritis (OA) often leads to pain and functional limitations, impacting work and daily life. Physical activity (PA) is an important part of the treatment. Wearable activity trackers (WATs) offer a novel approach to promote PA but could also aid in finding a sustainable PA level over time. The aim of this secondary analysis was to examine the effects of self-monitoring PA with a WAT on perceived joint function and health-related quality of life in people with hip and knee OA.

**Method:**

A two-armed cluster-randomized controlled trial (C-RCT) was conducted in southern Sweden including 160 individuals with hip or knee OA. The participants were cluster-randomized to a Supported Osteoarthritis Self-management Program (SOASP) with the addition of self-monitoring PA using a commercial WAT for 12 weeks (n = 86), or only the SOASP (*n* = 74). The outcomes include perceived joint function measured with HOOS/KOOS and health-related quality of Life (HRQoL) measured with EQ-5D-3L index and EQ VAS. Participants responded to the questionnaires at baseline and at follow-up after 3, 6 and 12 months. Statistical analyses involved linear mixed models, ANCOVA and paired t-test.

**Results:**

Participants with data from baseline and at least one follow-up were included in the analyses (*n* = 124). The analyses showed no statistically significant differences in changes between the groups in perceived joint function or HRQoL throughout the study period. Both groups improved in pain and symptoms, but the changes were small.

**Conclusion:**

The addition of WAT-use did not have any effect on perceived joint function or HRQoL. The participants’ relatively high baseline scores might have influenced the outcomes of this study. We suggest that future WAT-interventions target inactive people with OA and use devices that also captures other activities such as cycling or aquatic exercise.

**Trial registration:**

ClinicalTrials.gov, NCT03354091. Registered 15/11/2017.

## Background

Osteoarthritis (OA) is a common joint disorder that often results in pain, functional impairment and restricted participation in work and leisure activities [1–3]. The hip and knee joints are frequently affected by OA, potentially limiting ambulatory- and other physical activities [4]. Physical activity (PA) is recommended as an important element in the first-line treatment of hip and knee OA and may reduce pain, improve function and health-related quality of life (HRQoL) for individuals with OA [5–7]. Although regular PA may be particularly important for people with OA, previous research has suggested that people with OA are insufficiently physically active [8]. Joint pain has been suggested as one important barrier for people with OA to engage in PA [9]. Finding the optimal level of PA over time might also be difficult according to the results from a qualitative study [10].

Interventions targeting PA promotion in OA and chronic pain populations have shown limited effectiveness in the short term and no long-term effects [11–13]. Interventions incorporating supervised exercise appear more successful, albeit requiring substantial healthcare resources [14]. Given that OA is a chronic, non-fatal condition, self-management plays a vital and suitable role in this treatment [15]. In Sweden, a Supported Osteoarthritis Self-management Program (SOASP) is provided as the first-line treatment of patients with hip, knee and hand OA [16]. The SOASP combines information about OA with exercise, either individually or in group settings.

A relatively new and promising addition to PA interventions incorporating self-management is the utilisation of wearable activity trackers (WATs) [17]. WATs, produced by various companies, include features such as step counting, distance measurement, heart rate monitoring and sleep tracking [18]. These devices are often connected to mobile applications (apps) where users can set activity goals, receive feedback, and monitor their PA. Self-monitoring, receiving feedback and setting PA goals are examples of behaviour change techniques that have demonstrated effectiveness in interventions promoting PA [19, 20]. However, to self-monitor PA might also help individuals with OA to find a sustainable level of PA over time to effectively manage symptoms. In a qualitative study within this project, participants expressed that they used the WAT to identify an ‘optimal’ number of steps per day to avoid worsening of pain and other symptoms [21]. Therefore, in this study, we wanted to investigate the effect of WAT-use on the other outcomes, perceived joint function, joint-related pain, and HRQoL. The effects of WAT-use on work ability, PA and work productivity has been investigated in a prior study within this project [20].

To our knowledge, no prior studies have investigated the effect of WAT-use on joint function and health in people with OA. Hence, the aim of this secondary analysis was to examine the effects of adding self-monitoring PA with a WAT to the SOASP on perceived joint function and HRQoL in people with hip and knee OA.

## Methods

### Design and setting

We conducted a two-armed cluster-randomised controlled trial (C-RCT) in southern Sweden between 2017 and 2020 investigating the effects of adding self-monitoring of PA with a WAT to participating in the SOASP, compared to the SOASP alone. The trial was reported in clinical trials, 15/11/2017 (No: NCT03354091) [22]. The intervention showed no statistically significant effect on work ability, PA and work productivity [20]. This study is a secondary analysis of the C-RCT.

Participants in both groups in the C-RCT took part of the SOASP, a structured program mainly provided in primary healthcare to OA patients [16]. While the structural aspects of the SOASP may vary between healthcare centres or caregivers, the content and extent are generally similar. Participants received at least a minimum level of the SOASP, including two or three group lectures about osteoarthritis, self-care, and exercise. These lectures were often complemented by a period of supervised exercise.

### Participants and recruitment

**Fig. 1:**
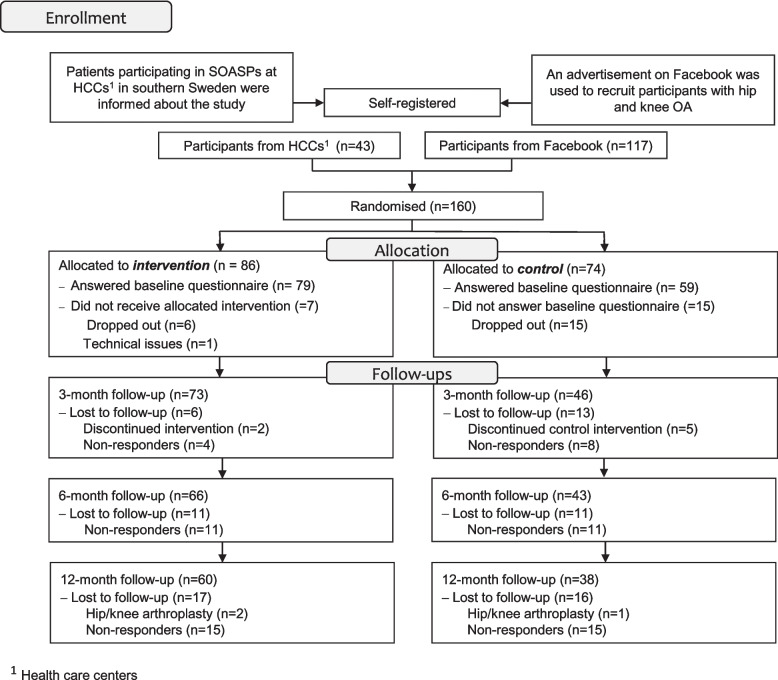
Enrolment and study process

Potential participants were recruited between 2017 and 2019 from healthcare centres or through advertisement on Facebook. Inclusion criteria were having hip and/or knee osteoarthritis (as diagnosed by a physician or physiotherapist), working ≥ 50% (20 h./week), aged between 18–67 years (working age), able to participate in PA and proficient in Swedish. Additionally, participants needed access to a smartphone and be able to wear a wrist-worn activity tracker for twelve consecutive weeks. A flowchart illustrating the recruitment and data collection process is presented in Fig. 1.

### Intervention

In addition to the SOASP, participants in the intervention group received a wrist-worn WAT (Fitbit Flex 2) and were instructed to self-monitor PA for twelve weeks. The WAT measures steps, distance and ‘active minutes’ [23]. The ‘active minutes’ is, according to the manufacturer, equivalent to MVPA [24]. The device is connected to a mobile app, allowing users to monitor their activity. A pre-defined step goal of 7,000 steps per day was set for all participants, with daily app usage encouraged. All participants in the intervention groups met with EÖ and received the Fitbit. They were aided in installing the Fitbit app and synchronizing the device to the participant’s app. They were asked to wear the Fitbit for twelve weeks, from morning until bedtime. The default activity goal of 10,000 steps per day was changed to 7,000 to make it more achievable for the participants. All other default activity goals (distance, calories burned and bouted active minutes) in the app were unchanged. Participants received prompts and feedback from the Fitbit app about their PA achievements by default.

### Data collection and outcome variables

An online survey was sent to the participants at baseline, before the intervention started and at follow-up after three, six and twelve months. The survey included questions on sociodemographic variables and patient-reported outcome measures for the outcome variables. The preceding paper analysed and reported work ability, PA level and work productivity. In this paper, we analysed perceived joint function and HRQoL.

Joint function was assessed using the Hip disability and Osteoarthritis Outcome Score (HOOS) or Knee injury and Osteoarthritis Outcome Score (KOOS) depending on the most affected joint [25, 26]. HOOS/KOOS consists of five subscales: Pain, Symptoms, Activities of Daily Living (ADL), Sport and Recreation function (Sport/Rec.) and joint-related Quality of Life (QoL). The subscales consists of items with a Likert scale and each subscale is calculated as a score between 0 to 100 where 0 indicates extreme hip or knee problems and 100 indicates no hip or knee problems [26, 27]. The instruments have demonstrated adequate psychometric qualities in OA populations [28, 29].

The Euroqol instrument EQ-5D-3L was used to measure self-rated HRQoL. The instrument comprises a descriptive system with five dimensions (mobility, self-care, usual activities, pain/discomfort and anxiety/depression) and a visual analogue scale (EQ VAS) [30]. The results for the five dimensions can be combined to a number that reflects the HRQoL while the EQ VAS provides a quantitative measure from 0 to 100 where 0 represent ‘Worst imaginable health state’ and 100 represent the ‘Best imaginable health state’. The validity and reliability of these instruments have been established [31].

### Sample size

A sample size calculation was carried out in the prior paper [20] using the primary outcome variable work ability, resulting in approximately 80 participants per group.

### Randomisation

Each SOASP group was considered a cluster and randomly allocated to either the control or intervention group. Recruitment and randomization occurred between October 2017 and May 2019, with a randomisation plan (1:1) generated from randomization.com using seven blocks. Sealed envelopes (n = 125) were used to cluster-randomise the SOASP groups, with 63 groups allocated to control and 62 to intervention. Neither authors nor participants were blinded after allocation to control or intervention.

### Data analyses

IBM SPSS Statistics version 28 was used for the analyses [32]. Participant characteristics were presented as mean (standard deviation (SD)) or proportions. Descriptive results for baseline and follow-ups were presented as mean (SD). A linear mixed model [33] was employed to investigate the effect of group allocation (intervention/control) on perceived joint function and HRQoL over the three, six, and twelve-month follow-ups from baseline. Both group and time were included as fixed factors in the analysis, with the interaction term Group*Time added to assess differences in the patterns of change (effects) between the two groups.

Differences in scores from baseline to each follow-up were calculated for each group. The differences between the groups were compared using analysis of covariance (ANCOVA) with adjustments for baseline scores [34]. Mean adjusted difference and 95% confidence intervals (CI) were presented. Changes *within* each group (baseline to three, six respectively twelve month follow-up) were analysed using paired t-test.

## Results

In the final analyses, only participants with data from baseline and at least one follow-up were included, 124 participants were included with 74 in the intervention group and 50 in the control group. The mean age for the total sample were 55.8 (5.7) years, ranging from 38 to 65 years. The majority were women, had postsecondary education and rated the knee as the most affected joint. Table 1 presents participants’ baseline characteristics. Additional characteristics has also been presented in previous publication [20].

**Table 1:** Participants' baseline characteristics (*n* = 124)

Group	Intervention (*n* = 74)	Control (*n* = 50)
**Age (years), mean (SD)**	56.7 (5.3)	54.5 (6.2)
**Sex, % (n)**		
Female	87.8 (65)	76.0 (38)
Male	12.2 (9)	24.0 (12)
**Married or living with partner, % (n)**	75.7 (56)	74.0 (37)
**Most affected joint, % (n)**		
Hip	21.6 (16)	30 (15)
Knee	78.4 (58)	70 (35)
**Education (postsecondary), % (n)**	67.6 (50)	62.0 (31)
**Regular usage of a WAT during the last three months before the intervention, % (n)**^**a**^	41.7 (30)	36.7 (18)
**Present physical activity level compared to before OA, % (n)**^**a**^		
More physically active	10.8 (8)	14.0 (7)
Less physically active	55.4 (41)	50.0 (25)
Equally physically active	32.4 (24)	36.0 (18)

*SD* Standard deviation, *WAT* Wearable activity tracker, *OA* Osteoarthritis

^a^ Results are presented as valid percent

### Perceived joint function

The HOOS/KOOS subscale ADL covering walking, grocery shopping, sock removal etc., had the highest mean (SD) score at baseline for the total sample, 69.8 (19.7). Baseline mean scores were 56.9 (20.5) for subscale Pain, 52.5 (20.6) for subscale Symptoms, and 42.6 (18.6) for subscale QoL. The subscale Sport/Rec. had the lowest (worst) mean score at 31.6 (24.9) indicating difficulties with activities such as jumping, running, and squatting. Table 2 presents mean scores (SD) for all measurements in both groups.

**Table 2:** Joint function and health-related quality of life at baseline and follow-ups. Changes within and differences between the groups from baseline to follow-ups

	Intervention		Control		Between group differences *(Intervention – Control)*	
**Outcome**	Mean (SD)	Adj.* change from baseline [95% CI]	Mean (SD)	Adj.* change from baseline [95% CI]	Adj.* difference [95% CI]	*p*
**HOOS/KOOS**						
*** Pain***						
Baseline	57.7 (20.2)	n/a	55.8 (21.0)	n/a	n/a	
3-month FU	65.2 (21.6)	7.5 [4.2, 10.7]	60.5 (21.1)	4.1 [0.05, 8.2]	3.4 [-1.8, 8.6]	*0.20*
6-month FU	63.3 (21.2)	5.3 [2.0, 8.7]	64.7 (19.0)	7.0 [2.9, 11.2]	-1.7 [-7.0, 3.7]	*0.53*
12-month FU	64.5 (22.6)	5.8 [1.8, 9.8]	64.8 (22.0)	6.7 [1.7, 11.8]	-0.9 [-7.4, 5.6]	*0.78*
*** Symptoms***						
Baseline	53.5 (20.6)	n/a	50.9 (20.7)	n/a	n/a	
3-month FU	58.3 (20.1)	5.1 [1.8, 8.4]	53.7 (18.9)	3.4 [-0.8, 7.5]	1.7 [-3.6, 7.0]	*0.53*
6-month FU	56.0 (21.8)	2.5 [-1.3, 6.3]	56.5 (22.2)	4.0 [-0.6, 8.7]	-1.5 [-7.5, 4.5]	*0.62*
12-month FU	57.9 (22.8)	4.3 [-0.5, 9.1]	56.8 (20.2)	5.4 [-0.6, 11.4]	-1.1 [-8.7, 6.6]	*0.79*
*** ADL***						
Baseline	70.1 (20.0)	n/a	69.4 (19.6)	n/a	n/a	
3-month FU	71.4 (21.5)	1.3 [-1.2, 3.8]	68.9 (20.8)	0.6 [-2.6, 3.7]	0.8 [-3.3, 4.8]	*0.71*
6-month FU	70.3 (22.3)	-0.6 [-3.5, 2.4]	74.3 (18.9)	3.4 [-0.3, 7.0]	-4.0 [-8.7, 0.8]	*0.10*
12-month FU	71.0 (22.6)	-1.0 [-4.6, 2.7]	71.3 (20.5)	0.9 [-3.6, 5.5]	-1.9 [-7.8, 3.9]	*0.52*
*** Sport/Rec***						
Baseline	30.3 (25.2)	n/a	33.5 (24.7)	n/a	n/a	
3-month FU	32.3 (25.8)	2.5 [-1.9, 6.9]	30.8 (24.9)	-0.1 [-5.7, 5.4]	2.6 [-4.5, 9.7]	*0.47*
6-month FU	32.7 (27.5)	0.4 [-4.8, 5.6]	34.7 (27.8)	1.0 [-5.3, 7.4]	-0.7 [-8.8, 7.5]	*0.87*
12-month FU	36.0 (29.7)	3.0 [-3.1, 9.2]	32.2 (25.8)	0.5 [-7.1, 8.1]	2.5 [-7.3, 12.3]	*0.61*
*** QoL***						
Baseline	43.2 (18.7)	n/a	41.9 (18.5)	n/a	n/a	
3-month FU	45.4 (20.7)	2.6 [-0.5, 5.6]	44.8 (20.6)	3.2 [-0.5, 7.0]	-0.7 [-5.5, 4.2]	*0.79*
6-month FU	46.4 (21.3)	2.6 [-0.9, 6.1]	47.5 (22.4)	4.4 [0.1, 8.7]	-1.8 [-7.3, 3.8]	*0.53*
12-month FU	46.4 (20.8)	1.8 [-2.4, 6.0]	48.4 (20.3)	4.7 [-0.6, 9.9]	-2.8 [-9.6, 3.9]	*0.40*
**EQ-5D-3L**						
Baseline	0.78 (0.09)	n/a	0.77 (0.07)	n/a	n/a	
3-month FU	0.79 (0.08)	0.01 [-0.01, 0.02]	0.78 (0.09)	0.01 [-0.01, 0.03]	-0.00 [-0.03, 0.02]	*0.84*
6-month FU	0.79 (0.08)	0.00 [-0.01, 0.02]	0.78 (0.09)	0.01 [-0.01, 0.03]	-0.00 [-0.03, 0.02]	*0.79*
12-month FU	0.80 (0.09)	0.01 [-0.02, 0.03]	0.81 (0.11)	0.03 [0.01, 0.06]	0.03 [-0.06, 0.01]	*0.10*
**EQ VAS**						
Baseline	73.2 (17.6)	n/a	74.2 (17.9)	n/a	n/a	
3-month FU	73.8 (20.2)	0.7 [-3.1, 4.5]	74.1 (19.2)	-0.4 [-5.2, 4.3]	1.1 [-5.0, 7.2]	*0.72*
6-month FU	73.2 (18.5)	0.6 [-4.0, 2.7]	77.3 (17.3)	1.7 [-2.4, 5.8]	-2.3 [-7.6, 2.9]	*0.38*
12-month FU	73.8 (18.2)	-0.23 [-3.8, 3.3]	75.6 (19.2)	0.8 [-3.6, 5.2]	-1.0 [-6.6, 4.7]	*0.73*

Analysis of Covariance (ANCOVA) were performed to analyze changes within and differences between the groups

*SD* standard deviation, *CI* confidence interval; (a) n denotes the number of participants with analyzable data for each variable/subscale, *FU* follow-up, *HOOS* Hip disability and Osteoarthritis Outcome Score, *KOOS* Knee injury and Osteoarthritis Outcome Score, *ADL* Activities of Daily Living, *Rec.* Recreation, *QoL* Quality Of Life; *EQ-5D-3L* EuroQol five dimensions 3-level, *VAS* visual analogue scale

^*^Adjusted for baseline values

Linear mixed model analyses showed no statistically significant interaction (Group*Time) for any of the HOOS/KOOS subscales: Pain (*p* = 0.41), Symptoms (*p* = 0.91), ADL (*p* = 0.53), Sport/Rec. (p = 0.78) and QoL (*p* = 0.72). However, there was a statistically significant main effect regarding time for Pain (p = < 0.01) and Symptoms (p = < 0.01), indicating general improvement over time in both groups. The ANCOVA showed no statistically significant differences between the groups in terms of change in perceived joint function for any of the periods (baseline to three-, six- or twelve-month follow-up) (Table 2). Results from the paired t-test showed a statistically significant improvement between baseline and all three follow-ups for both groups regarding subscale *Pain (*p’s = < 0.01)*.* For subscale *Symptoms*, there was a statistically significant improvement for both groups between baseline and three month follow-up (p’s = < 0.04) but not for the other two follow-ups (p’s = > 0.07). There were also statistically significant differences for the control group in subscale *ADL* but only from baseline to 6-month follow-up (p = 0.05) and not for intervention group or the other periods (p’s = > 0.4). No other statistically significant differences were shown for the other HOOS/KOOS subscales (p’s = > 0.08).

### Health-related quality of life

The EQ-5D-3L index for the total sample at baseline were 0.78 (0.08) and the EQ VAS value were 73.6 (17.7). The linear mixed model analyses showed no statistically significant interaction (Group*Time) for neither the EQ-5D (*p* = 0.51) nor the EQ VAS (*p* = 0.74). Furthermore, there were no statistically significant main effects regarding time. The ANCOVA showed no statistically significant differences between the groups regarding change in HRQoL for any of the periods (baseline to three, six or twelve month follow-ups) (Table 2). The paired t-test showed a statistically significant change in EQ-5D-3L for the control group from baseline to twelve month follow-ups (p = < 0.01) but not for the intervention group or other periods (p’s = > 0.3).

## Discussion

This study reports the outcomes of a secondary analysis from a C-RCT investigating the effects of adding self-monitoring of PA with a WAT on self-reported joint function and HRQoL in individuals with hip and knee OA. No statistically significant differences were observed between the groups in terms of changes during the study period for any of the outcomes. Both groups improved regarding pain and symptoms, but the changes were smaller than the *minimal important change* which previously has been established for KOOS subscale *Pain* (12.2 points) and HOOS subscale *Pain* (11.8 points) [35].

Impairments related to function and pain are common among people with hip and knee OA [4]. This study investigates the interventions’ effect on joint related pain, symptoms, ADL, Sport/Rec. and QoL. As expected, participants had the lowest (worst) mean scores on the Sport/Rec. subscale and the highest (best) scores on the ADL subscale, reflecting more difficulties with activities such as *jumping* than *rising from bed*. These results are in line with a meta-analysis (focusing on knee OA only), reporting the highest scores on ADL and the lowest on Sport/Rec. [29]. Compared to the meta-analysis, this study presents similar results on subscale Symptoms but better mean scores on the other four KOOS subscales [29].

The groups did not differ significantly in changes regarding outcomes throughout the measurement period. Additionally, subscales ADL, Sport/Rec. and QoL scores remained relatively stable during the measurements with, in general, no statistically significant differences according to the paired t-test. The PA levels among the participants also seems to have been relatively stable and high throughout the intervention period according to previously analysed WAT-data (intervention group only) and PROMS [20, 36]. Both groups improved regarding subscale Pain during the study period, but the changes were smaller than the *minimal important change* identified for the HOOS/KOOS subscale Pain in a recent study [35]. Rienstra et al. [35] demonstrated that the HOOS/KOOS subscale Pain has adequate responsiveness in detecting *minimal important change* after joint arthroplasty but not after conservative treatment.

The average EQ-5D-3L index score for all participants in this study was 0.78, which is higher than other OA samples in Sweden reporting index scores between 0.65–0.67 [37, 38]. Shalhoub et al. investigated factors related to HRQoL in people with OA [39] and found a negative association between OA pain and HRQoL. Furthermore, they also found that individuals with a higher educational level had higher HRQoL [39]. In our study, most participants had post-secondary education, which might, in part, explain the relatively high EQ-5D-3L index and EQ-VAS scores. Participants mean scores on EQ-5D-3L and EQ-VAS remained stable throughout the 12-month study period, with no differences in changes between the groups. The change in EQ-5D-3L after participation in the SOASP has been investigated in previous research, where a slight increase from baseline to 3-month follow-up was reported [38]. Nevertheless, that increase was only half of what is considered the minimal important difference for EQ-5D-3L index score in the knee OA population (score 0.15) [40]. In our study, neither the SOASP alone nor the SOASP with the addition of self-monitoring PA with a WAT had any effect on HRQoL reaching the minimal important difference.

Both groups had slight improvements regarding joint-related pain and symptoms which may be related to their participation in the SOASP. In a previous study, reduced movement-related fear and increased OA self-efficacy were highlighted as mediators that improved pain and physical function in people with knee OA [41]. In line with this, Åkesson et al. [42] showed that OA patients had an increase in empowerment (facilitating self-efficacy and autonomy) after participating in the SOASP. However, the absence of a control group receiving no treatment prevents us from drawing such conclusions. Natural fluctuations and regression to the mean are additional plausible explanations for the observed results [43].

The use of WATs in research has increased drastically during the last decade and PA-level is the most common outcome in research concerning OA and other populations [44]. While insufficient PA can have negative health effects, excessive engagement in weight-bearing activities is not solely beneficial for people with OA [45, 46]. Qualitative research on patients’ perspectives has, in fact, shown that WATs may be used as a guide to help in optimising PA to avoid a worsening of pain [21, 47]. It is important to note that PA cannot cure OA or always alleviate symptoms but, for people with OA, being able to remain physically active and find a sustainable balance between load and recovery has other important health-enhancing effects [10].

The results in this study should be interpreted in light of some methodological limitations. While the randomised controlled trial design and the utilisation of well-established, reliable outcome measures are fundamental strengths, there are also several limitations that should be acknowledged. We used the EQ-5D-3L and not the EQ-5D-5L which could be seen as a limitation since the latter has shown better measurement properties [48]. However, this project was initiated already in 2017 and the systematic review comparing the three-level and five-level versions had not yet been published [48]. The recruitment of participants through Facebook might have introduced a selection bias since it could lead to an overrepresentation of younger, white women [49]. Participants were also highly physically active at baseline, about 40% already used a WAT and, as mentioned previously, their perceived joint function and HRQoL were higher than other OA cohorts [20, 36]. Another factor that might be important to take into consideration is that most of the participants had postsecondary education, indicating high socioeconomic status. In a systematic review with meta-analysis, Western et al. [50], demonstrated that digital PA interventions were not equally effective for people of low and high socioeconomic status. In this project, we may not have reached individuals that needed the intervention the most. Considering that the participants in this study were already highly physically active, the possibilities for improvement during the study period was limited. Additionally, all participants were assigned the uniform step goal of 7,000 steps which might have been suitable for some but not all participants. In a qualitative study within this project, some participants said that the WAT facilitated and optimised PA while others said that they were unable to reach the step goal due to pain [21]. They also expressed that the WAT used in this study were not able to adequately capture all types of PA, especially not activities such as bicycling or strength training, which was seen as a limitation. We would also like to highlight that there were a higher number of dropouts in the control group, especially at the start of the study before filling out the baseline questionnaire. The reasons for dropping out were mainly a lack of time, a change of mind, changed conditions at work or family but they may also have dropped out because they did not receive the intervention.

## Conclusion

This secondary analysis of a C-RCT showed no effects of adding self-monitoring PA with a WAT on perceived joint function and HRQoL in individuals with hip and knee OA. The participants in this study were already highly physically active which limits the possibilities for improvements. Emphasis on potential future WAT-interventions should be placed on reaching people that would benefit the most from the intervention. We would also recommend the use of a WAT that can capture different types of physical activities since some OA patients might benefit from doing less weight-bearing activities such as bicycling or aquatic exercise.

## Data Availability

The datasets and analyses used in this study are available from the corresponding author on reasonable request.

## Abbreviations

ADL: Activities of daily living
ANCOVA: Analysis of covariance
App: Application
CI: Confidence intervals
C-RCT: Cluster-Randomised controlled trial
EQ-5D-3L: European Quality of Life 5 Dimensions 3 Level Version
HOOS: Hip disability and Osteoarthritis Outcome Score
HRQoL: Health-Related Quality of Life
KOOS: Knee Injury and Osteoarthritis Outcome Score
MVPA: Moderate to vigorous physical activity
OA: Osteoarthritis
PA: Physical activity
QoL: Quality of life
SD: Standard deviation
SOASP: Supported Osteoarthritis Self-management Program
Sport/Rec.: Sport and Recreation function
WAT: Wearable activity tracker
WHO: World Health Organization

## References

[1] Clynes MA, Jameson KA, Edwards MH, Cooper C, Dennison EM. Impact of osteoarthritis on activities of daily living: does joint site matter? Aging Clin Exp Res. 2019;31(8):1049–56. 30903599 10.1007/s40520-019-01163-0 PMC6661019

[2] Kingsbury SR, Gross HJ, Isherwood G, Conaghan PG. Osteoarthritis in Europe: impact on health status, work productivity and use of pharmacotherapies in five European countries. Rheumatol Oxf. 2014;53(5):937–47. 10.1093/rheumatology/ket463 24489012

[3] Hunter DJ, Bierma-Zeinstra S. Osteoarthritis. Lancet. 2019;393(10182):1745–59. 31034380 10.1016/S0140-6736(19)30417-9

[4] Weigl M, Wild H. European validation of The Comprehensive International Classification of Functioning, Disability and Health Core Set for Osteoarthritis from the perspective of patients with osteoarthritis of the knee or hip. Disabil Rehabil. 2018;40(26):3104–12. 28911242 10.1080/09638288.2017.1377295

[5] Bannuru RR, Osani MC, Vaysbrot EE, Arden NK, Bennell K, Bierma-Zeinstra SMA, et al. OARSI guidelines for the non-surgical management of knee, hip, and polyarticular osteoarthritis. Osteoarthritis Cartilage. 2019;27(11):1578–89. 31278997 10.1016/j.joca.2019.06.011

[6] Si J, Sun L, Li Z, Zhu W, Yin W, Peng L. Effectiveness of home-based exercise interventions on pain, physical function and quality of life in individuals with knee osteoarthritis: a systematic review and meta-analysis. J Orthop Surg. 2023;18(1):503. 10.1186/s13018-023-04004-z PMC10351144 37461112

[7] Kraus VB, Sprow K, Powell KE, Buchner D, Bloodgood B, Piercy K, et al. Effects of Physical Activity in Knee and Hip Osteoarthritis: A Systematic Umbrella Review. Med Sci Sports Exerc. 2019;51(6):1324–39. 31095089 10.1249/MSS.0000000000001944 PMC6527143

[8] Chang AH, Song J, Lee J, Chang RW, Semanik PA, Dunlop DD. Proportion and associated factors of meeting the 2018 Physical Activity Guidelines for Americans in adults with or at risk for knee osteoarthritis. Osteoarthritis Cartilage. 2020;28(6):774–81. 10.1016/j.joca.2020.03.007 PMC7261619 32200050

[9] Kanavaki AM, Rushton A, Efstathiou N, Alrushud A, Klocke R, Abhishek A, et al. Barriers and facilitators of physical activity in knee and hip osteoarthritis: a systematic review of qualitative evidence. BMJ Open. 2017;7(12):e017042. 29282257 10.1136/bmjopen-2017-017042 PMC5770915

[10] Kanavaki AM, Rushton A, Hale E, Klocke R, Abhishek A, Duda JL. Physical activity, sedentary behaviour and well-being: experiences of people with knee and hip osteoarthritis. Psychol Health. 2024;39(8):1023–41. 36184868 10.1080/08870446.2022.2126473

[11] Bell EC, Wallis JA, Goff AJ, Crossley KM, O’Halloran P, Barton CJ. Does land-based exercise-therapy improve physical activity in people with knee osteoarthritis? A systematic review with meta-analyses. Osteoarthritis Cartilage. 2022;30(11):1420–33. 35970256 10.1016/j.joca.2022.07.008

[12] Williamson W, Kluzek S, Roberts N, Richards J, Arden N, Leeson P, et al. Behavioural physical activity interventions in participants with lower-limb osteoarthritis: a systematic review with meta-analysis. BMJ Open. 2015;5(8):e007642. 26260348 10.1136/bmjopen-2015-007642 PMC4538274

[13] Fernandopulle S, Perry M, Manlapaz D, Jayakaran P. Effect of Land-Based Generic Physical Activity Interventions on Pain, Physical Function, and Physical Performance in Hip and Knee Osteoarthritis: A Systematic Review and Meta-Analysis. Am J Phys Med Rehabil. 2017;96(11):773–92. 28323761 10.1097/PHM.0000000000000736

[14] Jordan JL, Holden MA, Mason EE, Foster NE. Interventions to improve adherence to exercise for chronic musculoskeletal pain in adults. Cochrane Database Syst Rev. 2010;2010(1):CD005956. 20091582 10.1002/14651858.CD005956.pub2 PMC6769154

[15] Grady PA, Gough LL. Self-Management: A Comprehensive Approach to Management of Chronic Conditions. Am J Public Health. 2014;104(8):e25-31. 24922170 10.2105/AJPH.2014.302041 PMC4103232

[16] Thorstensson CA, Garellick G, Rystedt H, Dahlberg LE. Better Management of Patients with Osteoarthritis: Development and Nationwide Implementation of an Evidence-Based Supported Osteoarthritis Self-Management Programme. Musculoskeletal Care. 2015;13(2):67–75. 25345913 10.1002/msc.1085

[17] Brickwood KJ, Watson G, O’Brien J, Williams AD. Consumer-Based Wearable Activity Trackers Increase Physical Activity Participation: Systematic Review and Meta-Analysis. JMIR MHealth UHealth. 2019;7(4):e11819. 30977740 10.2196/11819 PMC6484266

[18] Hoy MB. Personal Activity Trackers and the Quantified Self. Med Ref Serv Q. 2016;35(1):94–100. 26794199 10.1080/02763869.2016.1117300

[19] Mercer K, Li M, Giangregorio L, Burns C, Grindrod K. Behavior Change Techniques Present in Wearable Activity Trackers: A Critical Analysis. JMIR MHealth UHealth. 2016;4(2):e40. 27122452 10.2196/mhealth.4461 PMC4917727

[20] Östlind E, Eek F, Stigmar K, Sant’Anna A, Hansson EE. Promoting work ability with a wearable activity tracker in working age individuals with hip and/or knee osteoarthritis: a randomized controlled trial. BMC Musculoskelet Disord. 2022;23(1):112. 35114983 10.1186/s12891-022-05041-1 PMC8812043

[21] Östlind E, Ekvall Hansson E, Eek F, Stigmar K. Experiences of activity monitoring and perceptions of digital support among working individuals with hip and knee osteoarthritis - a focus group study. BMC Public Health. 2022;22(1):1641. 36042425 10.1186/s12889-022-14065-0 PMC9426251

[22] ClinicalTrials. gov. Clinical trials: Active@Work - Optimizing physical activity at work. 2020.

[23] Fitbit. Manual Fitbit Flex 2. [cited 2022 Jan 11]. Available from: https://staticcs.fitbit.com/content/assets/help/manuals/manual_flex_2_en_US.pdf.

[24] Fitbit. Fitbit help article. 2020 [cited 2022 Aug 18]. Available from: https://help.fitbit.com/articles/en_US/Help_article/1379.htm.

[25] Roos EM, Roos HP, Lohmander LS, Ekdahl C, Beynnon BD. Knee Injury and Osteoarthritis Outcome Score (KOOS)–development of a self-administered outcome measure. J Orthop Sports Phys Ther. 1998;28(2):88–96. 9699158 10.2519/jospt.1998.28.2.88

[26] Nilsdotter AK, Lohmander LS, Klässbo M, Roos EM. Hip disability and osteoarthritis outcome score (HOOS)–validity and responsiveness in total hip replacement. BMC Musculoskelet Disord. 2003;4:10–10. 12777182 10.1186/1471-2474-4-10 PMC161815

[27] Roos EM, Lohmander LS. The Knee injury and Osteoarthritis Outcome Score (KOOS): from joint injury to osteoarthritis. Health Qual Life Outcomes. 2003;1:64–64. 14613558 10.1186/1477-7525-1-64 PMC280702

[28] Thorborg K, Roos EM, Bartels EM, Petersen J, Hölmich P. Validity, reliability and responsiveness of patient-reported outcome questionnaires when assessing hip and groin disability: a systematic review. Br J Sports Med. 2010;44(16):1186. 19666629 10.1136/bjsm.2009.060889

[29] Collins NJ, Prinsen CAC, Christensen R, Bartels EM, Terwee CB, Roos EM. Knee Injury and Osteoarthritis Outcome Score (KOOS): systematic review and meta-analysis of measurement properties. Osteoarthritis Cartilage. 2016;24(8):1317–29. 27012756 10.1016/j.joca.2016.03.010

[30] Rabin R, de Charro F. EQ-5D: a measure of health status from the EuroQol Group. Ann Med. 2001;33(5):337–43. 11491192 10.3109/07853890109002087

[31] Cheng LJ, Tan RLY, Luo N. Measurement Properties of the EQ VAS Around the Globe: A Systematic Review and Meta-Regression Analysis. Value Health J Int Soc Pharmacoeconomics Outcomes Res. 2021;24(8):1223–33. 10.1016/j.jval.2021.02.003 34372988

[32] IBM Corp. Released 2021. IBM SPSS Statistics for Windows, Version 28.0. Armonk, NY: IBM Corp. 2021. [cited 2023 Jun 19]. Available from: https://www.ibm.com/products/spss-statistics.

[33] Ritz C. Statistical Analysis of Continuous Outcomes from Parallel-Arm Randomized Controlled Trials in Nutrition-a Tutorial. Eur J Clin Nutr. 2021;75(1):160–71. 32939044 10.1038/s41430-020-00750-z

[34] Van Breukelen GJ. ANCOVA versus change from baseline: more power in randomized studies, more bias in nonrandomized studies [corrected]. J Clin Epidemiol. 2006;59(9):920–5. 16895814 10.1016/j.jclinepi.2006.02.007

[35] Rienstra W, Stevens M, Blikman T, Bulstra SK, van den Akker-Scheek I. Responsiveness and interpretability of the pain subscale of the Knee and Hip Osteoarthritis Outcome Scale (KOOS and HOOS) in osteoarthritis patients according to COSMIN guidelines. PLoS ONE. 2023;18(11):e0293760. 37971978 10.1371/journal.pone.0293760 PMC10653527

[36] Östlind E, Sant’Anna A, Eek F, Stigmar K, Ekvall Hansson E. Physical activity patterns, adherence to using a wearable activity tracker during a 12-week period and correlation between self-reported function and physical activity in working age individuals with hip and/or knee osteoarthritis. BMC Musculoskelet Disord. 2021;22(1):450. 33992121 10.1186/s12891-021-04338-x PMC8126142

[37] Kiadaliri AA, Lamm CJ, de Verdier MG, Engström G, Turkiewicz A, Lohmander LS, et al. Association of knee pain and different definitions of knee osteoarthritis with health-related quality of life: a population-based cohort study in southern Sweden. Health Qual Life Outcomes. 2016;14(1):121. 27565135 10.1186/s12955-016-0525-4 PMC5002211

[38] Sturesdotter Åkesson K, Beckman A, Stigmar K, Sundén A, Ekvall HE. Physical activity and health-related quality of life in men and women with hip and/or knee osteoarthritis before and after a supported self-management programme – a prospective observational study. Disabil Rehabil. 2022;44(16):4275–83. 33761294 10.1080/09638288.2021.1900417

[39] Shalhoub M, Anaya M, Deek S, Zaben AH, Abdalla MA, Jaber MM, et al. The impact of pain on quality of life in patients with osteoarthritis: a cross-sectional study from Palestine. BMC Musculoskelet Disord. 2022;23(1):248. 35287651 10.1186/s12891-022-05207-x PMC8919689

[40] Devji T, Guyatt GH, Lytvyn L, Brignardello-Petersen R, Foroutan F, Sadeghirad B, et al. Application of minimal important differences in degenerative knee disease outcomes: a systematic review and case study to inform BMJ Rapid Recommendations. BMJ Open. 2017;7(5):e015587. 28495818 10.1136/bmjopen-2016-015587 PMC5777462

[41] Lawford BJ, Hinman RS, McManus F, Lamb KE, Egerton T, Keating C, et al. How Does Exercise, With and Without Diet, Improve Pain and Function in Knee Osteoarthritis? A Secondary Analysis of a Randomized Controlled Trial Exploring Potential Mediators of Effects. Arthritis Care Res. 2023;75(11):2316–27. 10.1002/acr.25140 PMC10952828 37128836

[42] Åkesson KS, Sundén A, Stigmar K, Fagerström C, Pawlikowska T, Ekvall HE. Enablement and empowerment among patients participating in a supported osteoarthritis self-management programme - a prospective observational study. BMC Musculoskelet Disord. 2022;23(1):555. 35676666 10.1186/s12891-022-05457-9 PMC9175380

[43] Englund M, Turkiewicz A. Pain in clinical trials for knee osteoarthritis: estimation of regression to the mean. Lancet Rheumatol. 2023;5(6):e309–11. 38251596 10.1016/S2665-9913(23)00090-5

[44] Li C, Chen X, Bi X. Wearable activity trackers for promoting physical activity: A systematic meta-analytic review. Int J Med Inf. 2021;152:104487. 10.1016/j.ijmedinf.2021.104487 34020170

[45] Dore DA, Winzenberg TM, Ding C, Otahal P, Pelletier JP, Martel-Pelletier J, et al. The association between objectively measured physical activity and knee structural change using MRI. Ann Rheum Dis. 2013;72(7):1170–5. 22896739 10.1136/annrheumdis-2012-201691

[46] Allen KD, Thoma LM, Golightly YM. Epidemiology of osteoarthritis. Osteoarthritis Cartilage. 2022;30(2):184–95. 34534661 10.1016/j.joca.2021.04.020 PMC10735233

[47] Leese J, Macdonald GG, Tran BC, Wong R, Backman CL, Townsend AF, et al. Using Physical Activity Trackers in Arthritis Self-Management: A Qualitative Study of Patient and Rehabilitation Professional Perspectives. Arthritis Care Res. 2019;71(2):227–36. 10.1002/acr.23780 30295430

[48] Buchholz I, Janssen MF, Kohlmann T, Feng YS. A Systematic Review of Studies Comparing the Measurement Properties of the Three-Level and Five-Level Versions of the EQ-5D. Pharmacoeconomics. 2018;36(6):645–61. 29572719 10.1007/s40273-018-0642-5 PMC5954044

[49] Whitaker C, Stevelink S, Fear N. The Use of Facebook in Recruiting Participants for Health Research Purposes: A Systematic Review. J Med Internet Res. 2017;19(8):e290. 28851679 10.2196/jmir.7071 PMC5594255

[50] Western MJ, Armstrong MEG, Islam I, Morgan K, Jones UF, Kelson MJ. The effectiveness of digital interventions for increasing physical activity in individuals of low socioeconomic status: a systematic review and meta-analysis. Int J Behav Nutr Phys Act. 2021;18(1):148. 34753490 10.1186/s12966-021-01218-4 PMC8576797

[51] World Medical Association. World Medical Association Declaration of Helsinki: ethical principles for medical research involving human subjects. JAMA. 2013;310(20):2191–4. 24141714 10.1001/jama.2013.281053

